# Raman and Photoluminescence Spectroscopy with a Variable Spectral Resolution

**DOI:** 10.3390/s21237951

**Published:** 2021-11-28

**Authors:** Ivan Pavić, Joško Šoda, Vlatko Gašparić, Mile Ivanda

**Affiliations:** 1 Department for Marine Electrical Engineering and Information Technologies, Faculty of Maritime Studies, Ruđera Boškovića 37, 21000 Split, Croatia; jsoda@pfst.hr; 2 Laboratory for Molecular Physics and Synthesis of New Materials, Ruđer Bošković Institute, Bijenička Cestra 54, 10000 Zagreb, Croatia; vlatko.gasparic@irb.hr

**Keywords:** Raman spectrometer, photoluminescence, spectral resolution, zoom lens

## Abstract

Raman and photoluminescence (PL) spectroscopy are important analytic tools in materials science that yield information on molecules’ and crystals’ vibrational and electronic properties. Here, we show results of a novel approach for Raman and PL spectroscopy to exploit variable spectral resolution by using zoom optics in a monochromator in the front of the detector. Our results show that the spectral intervals of interest can be recorded with different zoom factors, significantly reducing the acquisition time and changing the spectral resolution for different zoom factors. The smallest spectral intervals recorded at the maximum zoom factor yield higher spectral resolution suitable for Raman spectra. In contrast, larger spectral intervals recorded at the minimum zoom factor yield the lowest spectral resolution suitable for luminescence spectra. We have demonstrated the change in spectral resolution by zoom objective with a zoom factor of 6, but the perspective of such an approach is up to a zoom factor of 20. We have compared such an approach on the prototype Raman spectrometer with the high quality commercial one. The comparison was made on ZrO_2_ and TiO_2_ nanocrystals for Raman scattering and Al_2_O_3_ for PL emission recording. Beside demonstrating that Raman spectrometer can be used for PL and Raman spectroscopy without changing of grating, our results show that such a spectrometer could be an efficient and fast tool in searching for Raman and PL bands of unknown materials and, thereafter, spectral recording of the spectral interval of interest at an appropriate spectral resolution.

## 1. Introduction

In various scientific fields of research, it is necessary to analyze materials’ structural and electronic properties. Scientists have developed various qualitative and quantitative analytic methods, including Raman and photoluminescence (PL) spectroscopies [1,2,3]. The Raman effect is an inelastic photon scattering process in which a transfer of energy between the light and the system corresponds to the energy of a particular vibrational state of the material. The result of Raman scattering is a Raman spectrum, which is unique to the individual molecule or crystal, and is therefore considered and studied as a “fingerprint” of the specific material [4,5]. In recent times, since the signal of Raman scattering is very weak, a number of new techniques for Raman scattering enhancement have been developed [6,7,8,9,10,11,12,13].When the luminescent molecule is excited by interaction with a photon of electromagnetic radiation, fluorescence or phosphorescence may occur. However, if the electromagnetic energy is released immediately, it is a fluorescence process; and if the release of electromagnetic energy is delayed, it is a phosphorescence process. Both fluorescence and phosphorescence are two forms of PL [14,15].

Spectra obtained by the Raman spectroscopy are usually accompanied by the broad background dominated by the intrinsic PL from the sample [16]. In the case of a moderate or weak PL signal, it could be recorded with a Raman spectrometer as a superposition on the Raman spectrum. However, if the PL signal is stronger and spectrally broader because of larger cross section of the fluorescence process, the recording process consists of a lot of spectral intervals (defined by the physical size of the CCD) combined into the single superimposed PL and Raman spectrum. This process is time-consuming and could produce unwanted artifacts depending on how the spectral intervals are combined into the single PL spectrum. In the case of a very strong PL signal, it completely masks the Raman signal [17,18]. Therefore, the Raman spectrometer could be a great tool for providing information on the PL phenomenon. Besides, when the Raman and PL spectral information [19,20,21,22] are both taken from the same material or even of its microscopic part, they could provide complementary information on the material’s vibrational and electronic properties. Additionally, combining Raman and PL techniques helps to detect specific structural features of complex chemical and biological samples [23,24]. Raman and PL spectroscopy is used in biology, pharmaceuticals, chemistry, environmental analysis, marine, electrical engineering, geology, and many other fields [25,26,27,28,29,30,31,32,33]. Several attempts [34,35,36,37,38] have been made to build cost-effective laboratory-based and portable Raman spectrometers of low spectral resolution. Their main purpose is to record Raman spectra for fast inspection of materials. However, a middle- or high-resolution Raman spectrometer is needed to observe the splitting of narrow spectral lines and/or small changes in the shift and width of the observed spectral lines. Furthermore, due to weak Raman scattering, the most sensitive detectors are used in many commercial laboratory-based Raman spectrometers. These detectors could also be very useful in detecting PL signal, even for the cases of a moderate or weak PL signal which is below the threshold of detection of the conventional PL spectrometers.

This paper presents a novel concept for developing a Raman spectrometer with a variable spectral resolution by implementing zoom optics into the monochromator of the spectrometer. Within this approach, it is possible to measure both the large spectral intervals and fast low resolution acquisitions on the one end, and high-resolution, detailed spectral images on the other end. The large spectral intervals are obtained with the smallest zoom factor (ZF) of the inserted optics. This is suitable for recording PL spectra, and the fast inspection of the whole Raman spectrum since the same amount of photons is distributed over a smaller number of pixels on the CCD. For the acquisition of detailed Raman spectra, a high spectral resolution is required. This is usually achieved by reducing the slit width, increasing the number of grooves on diffraction grating, and increasing the focal length of the focusing mirror to the detector. Contrary to the usual solutions, the degree of spectral resolution in the proposed spectrometer can be varied by the ZF of the focusing zoom lens to the detector [39,40]. 

## 2. Materials and Methods

The prototype of the instrumental setup and its application in recording Raman and PL spectra are described and compared with the complementary information acquired on the same samples by the reference Raman spectrometer to validate the proposed concept. The proposed hardware for recording Raman and PL spectra, the samples used for measurements, and the results for validation of the variable spectral resolution are described.

### 2.1. Instrumental Setup

**Figure 1:**
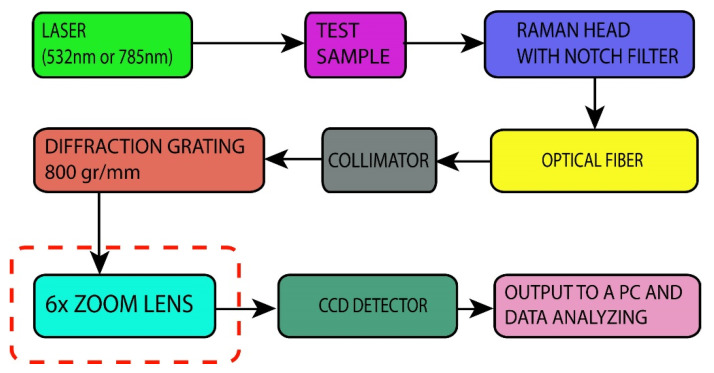
Schematic diagram of the proposed Raman spectrometer with an additional zoom lens component (red dashed rectangle) based on a typical Raman spectrometer configuration.

The schematic diagram of the proposed Raman spectrometer with the variable spectral resolution is shown in Figure 1, together with the implemented additional zoom lens component.

**Figure 2:**
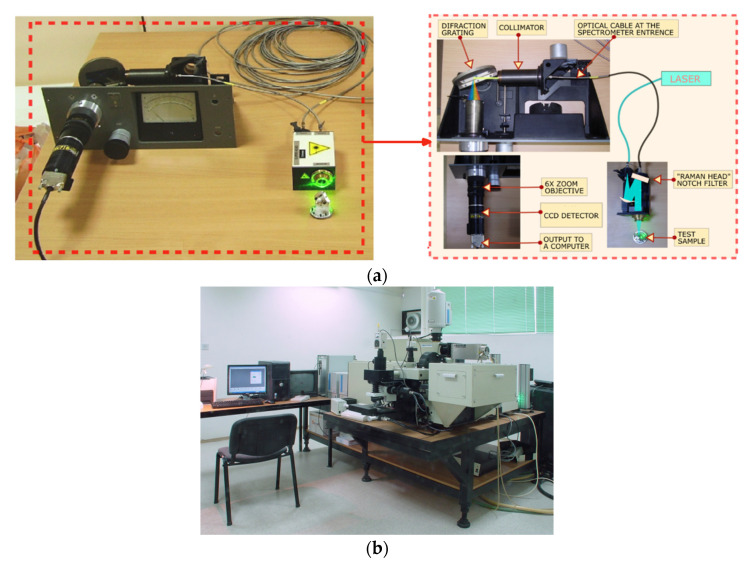
Assembled prototype of the proposed Raman spectrometer (**a**) and the reference Raman spectrometer JY-T64000 (**b**).

In the proposed Raman spectrometer, contrary to the classical approach, the second collimating mirror that focuses Raman or the PL signal to the CCD detector is replaced with a zoom lens, as shown in Figure 1. For such a case, we used a 6× zoom Fujinon-TV lens, f/1.6, with a variable focus f_co2_ = 14–84 mm. The working prototype assembly is shown in Figure 2a. It is based on commercial, off-the-shelf, and custom-built components. The excitation laser of wavelength λ = 532 nm was a DPSS laser (CNI, model DHL-W 532–500 mW). It was coupled to the off-the-shelf Raman head with a notch filter (that blocks Rayleigh scattered light) via a multimode optical fiber (ThorLabs, 0.10 NA, d = 25μm, ThorLabs GmbH, Bergkirchen, Germany). An additional 785-nm laser (CNI, model FC-D-785–450 mW) was used for the wavelength calibration. The same multimode fiber was used for the incoming laser excitation of the sample as well as the signal carrier to the custom-built spectrometer. The optical fiber’s exit is placed in the focus of the first collimating lens (f_co1_ = 150 mm), which provides a parallel light beam of the signal after the exit from the optical fiber. This parallel light beam falls onto the diffraction grating with 800 grooves/mm. After grating, the dispersed beam is focused onto the CCD by using a zoom lens. In our case, the CCD detector was Starlight Xpress, model MX716, with 752 × 580 pixels of size 8.6 × 8.3 um. 

To validate the principles of the proposed spectrometer, a high-grade laboratory Raman spectrometer (Horiba, JY-T64000. HORIBA Jobin Yvon GmbH, Bensheim, Germany), shown in Figure 2b, was used to compare the recorded spectra. Depending on the requirement, it can operate as a single, a double, or a triple monochromator spectrometer with the double foremonochromator usable as a subtractive filter, or as a double additive dispersive system for the ultimate resolution. In the single monochromator mode, it has a spectral resolution of 0.55 cm^−1^. It contains liquid-nitrogen-cooled CCD (Cryogenic, Symphony I, HORIBA Jobin Yvon GmbH, Bensheim, Germany 1024 × 256 pixels, pixel size 26 × 26 μm), a 640-mm focal length monochromator, and a working spectral range from 400 to 1100 nm. The diffraction grating of the reference spectrometer was 1800 grooves/mm. To ensure the same conditions for the incoming Raman scattered photons in both spectrometers, after recording the spectra with a prototype spectrometer, the optical cable was detached from its entrance and attached to the entrance slit of the third (single) monochromator of the JY T64000 spectrometer. This way, the same Raman and PL signal was analysed on both spectrometers.

### 2.2. The Samples Analysed for Comparison

Three samples with a different spectral resolution requirements were used to test the prototype’s spectral zooming performance for Raman and PL spectra recording. The first two samples are powder forms of nanosized zirconium oxide (ZrO_2_) and titanium oxide (TiO_2_) crystals. These samples were used due to their strong and known Raman bands located at 335, 475, and 620 cm^−1^ for ZrO_2_ and 400, 520, and 640 cm^−1^ for TiO_2_. The third sample is a powder of alumina oxide (Al_2_O_3_) which has a strong and broad PL signal in the spectral interval of 500–850 nm. The samples were placed in separate transparent cuvettes for the measurements for both Raman spectrometers. 

### 2.3. Raman and PL Spectra

To record Raman and PL spectra, the wavelength interval on the CCD region of the proposed Raman spectrometer was first calibrated with two lasers (λ_green_ = 532 nm, λ_red_ = 785 nm), converting each pixel to the corresponding wavelength value. A 300–1200-nm spectral range was obtained at the minimum ZF, and 490–616 nm at the maximum ZF. After the calibration, a 532-nm excitation laser was used for all samples. The output power was calibrated so that the laser power incident on the sample surface was 5 mW, measured with a portable laser power meter (Coherent Laser Check Power Meter, Kvant Lasers UK Limited, Daventry, UK). The exposure time was set to 1 s for the Raman measurements and 3 s for the PL measurements for both the prototype and the reference spectrometer. The wavelength interval for the Raman measurement was set according to the ZrO_2_ and TiO_2_ spectral lines. 

For all Raman spectra acquired with the proposed systems, the data were processed using Matlab R2018. It needs to be pointed out that, by taking the median value of all pixels in the vertical axis and then normalizing it in the acquired spectra, the noise (such as hot pixels and salt-and-pepper noise) was eliminated. This resulted in one-dimensional spectra plotted as intensity vs. wave number. Equations (1) and (2) show the proposed system’s spectral resolution analytically. Equation (1) is a Gaussian function used to fit Raman bands, and Equation (2) shows a full width at half maximum (FWHM) [41,42] for the Stokes lines of the ZrO_2_ Raman spectra at a minimum and maximum ZF. The variable parameters were peak intensity A, the peak center position b, and the standard deviation of the Gaussian fit σ.

(1) $$y=Ae^{\frac{{\left(x-b\right)}^{2}}{2\sigma^{2}}}$$

(2) $$FHWM=2\sigma \sqrt{2ln2},$$

Assuming a Gaussian line profile [43], a reasonable approximation for the FWHM of the Raman lines is provided by the relationship:

(3) $$FWHM=\sqrt{{\left(\Delta {\;\tilde{\nu }}_{slit}\right)}^{2}+{\left(\Delta {\;\tilde{\nu }}_{resolution}\right)}^{2}+{\left(\Delta {\;\tilde{\nu }}_{line}\right)}^{2}}$$

where:

- $\Delta {\;\tilde{\nu }}_{slit}$ is the true Raman bandwidth;
- $\Delta {\;\tilde{\nu }}_{resolution}$ is the spectral resolution of the spectrometer;
- $\Delta {\;\tilde{\nu }}_{line}$ is the Raman line width.

## 3. Results and Discussion

Raman spectra of ZrO_2_ were recorded with a different ZF. Then, TiO_2_ and ZrO_2_ spectra were recorded with the maximum ZF and compared with Raman spectra of the same samples obtained with JY-T64000 spectrometer. Next, the spectral resolution for the different ZF was analytically calculated. Finally, a comparison of the PL spectrum of Al_2_O_3_ obtained with the proposed system and JY-T64000 was performed.

### 3.1. Raman Spectra

**Figure 3:**
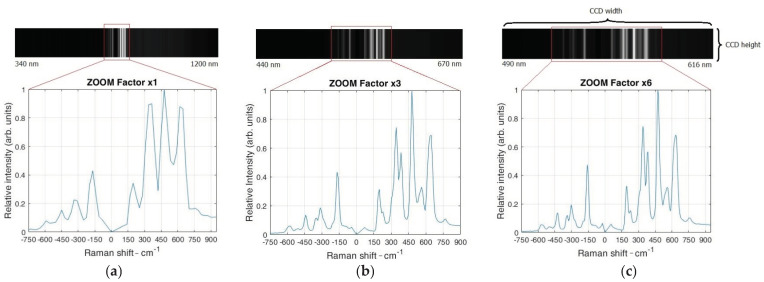
Acquired image over the CCD region and a Raman spectrum of a ZrO_2_ with different zoom factors, (**a**) for a ZF of 1×, (**b**) for 3×, and (**c**) maximum ZF of 6×.

The Raman spectra of ZrO_2_ acquired with the prototype Raman system with the different ZF are shown in Figure 3.

It has to be pointed out that recorded spectral lines of the used sample are shown on the top of each figure, and an intensity vs. Raman shift is shown on the bottom a spectre. Figure 3a shows the spectral interval over the entire CCD region at a ZF of 1× range, i.e., from 340 to 1200 nm. In this case, only a few spectral lines appear around the expected location of the 532-nm laser. The spectrum shows few anti-Stokes lines and three strong Stokes lines at 337.2, 477.6, and 618.1 cm^−1^. However, more spectral lines appear when the ZF increases three times, as shown in Figure 3b. The spectral interval over the CCD range is now from 440 to 670 nm. Moreover, it can be observed that the obtained spectra have additional “hidden” Stokes lines at wavelengths 337.2 and 477.6 cm^−1^ due to the increased spectral resolution. Furthermore, if the ZF is set to the maximum value of 6× (Figure 3c), it can be observed that the spectral interval becomes even narrower across the CCD region, measuring only 490–616 nm. It has to be pointed out that the spectral lines at this ZF are more distinct than at a lower ZF. Therefore, by increasing the ZF, the total spectral interval over the CCD region reduces and the spectral resolution increases, which is evident from the appearance of new lines accompanied with the line narrowing. 

**Figure 4:**
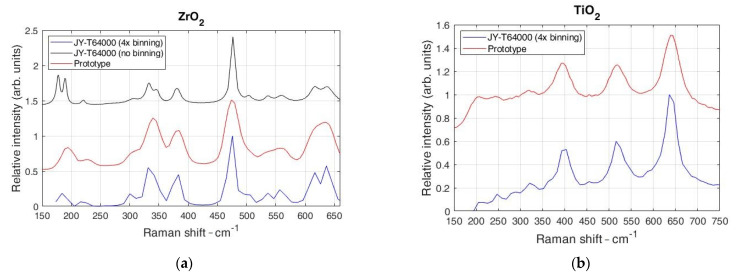
Comparison of the Raman spectra of ZrO_2_ (**a**) and TiO_2_ (**b**) using the proposed spectrometer with a reference spectrometer. Note that the reference spectrometer spectra without binning at (**a**) does not match spectral dispersion of prototype spectrometer; therefore, binning is used.

Figure 4 shows the Raman spectra of TiO_2_ and ZrO_2_ in comparison with the reference Raman spectra.

The ZF was set to the maximum 6× magnification, and the spectral interval from 537 to 557 nm was recorded. From Figure 4, it can be seen that both spectrometers (prototype and the reference model, respectively) show similar spectra. However, in Figure 4a, on the left-hand side, the laboratory-grade spectrometer shows additional spectral lines at 552 and 630 cm^−1^ for ZrO_2_. This is due to the reference spectrometer’s high spectral resolution achieved with the long focal length and a minimum noise generated by deep-cooled CCD. However, for the TiO_2_ sample seen in Figure 4b, the Raman spectra match the reference spectrometer’s spectra, which is due to the strong Stokes peaks of a sample where additional “hidden” peaks do not exist. 

### 3.2. Spectral Resolution

The following equation gives the relationship between the reciprocal linear dispersion (D^−1^), the focal length (f), the order of the diffraction (n), and the distance between blazes (d).

(4) $$D^{-1}=\frac{d}{n*f}$$

By substituting the value of the focal length f = 84 mm for the maximal ZF, the order of the diffraction n = 1, and the distance between rulings d = 1/800 mm, in the above equation, the reciprocal linear dispersion (D^−1^) for the first-order diffraction is 14.9 nm/mm. The per-pixel width of this value is 0.128 nm/pixel. The spectral dispersion at the position of the Raman band at 483.5 cm^−1^, as shown in Figure 3c (546 nm wavelength of light), is 4.28 cm^−1^/pixel. The same calculation for the reference Raman spectrometer gives a dispersion of 0.756 cm^−1^/pixel, which is a 5.67 times smaller value. For this reason, in order to arrive at a similar spectral resolution, a binning factor of four times was used during recording by the reference Raman spectrometer.

The input slit also limits the spectral resolution. The input slit width of the proposed spectrometer was fixed and determined by the diameter of the coupled optical fiber core, which is 25 μm. At the maximal ZF of 6×, the total magnification of the monochromator is f_co2_/f_co1_ = 0.56, which gives us the image size of 14 μm of the fiber core at the CCD. From the Nyquist limit, it can be determined whether the CCD sensor can differentiate between the two neighboring spectral lines, i.e., three times the core size image on CCD. Therefore, in our case, the CCD sensor can differentiate between two adjacent spectral lines when their peaks are separated 42 μm on the CCD, which spectrally is 21 cm^−1^ for the wavelength of 546 nm. The difference of the peak positions observed as two humps (overlapping lines) in the spectral interval 520–580 cm^−1^ (shown in Figure 4a) is 28.2 cm^−1^, which is very close to the expected value.

**Figure 5:**
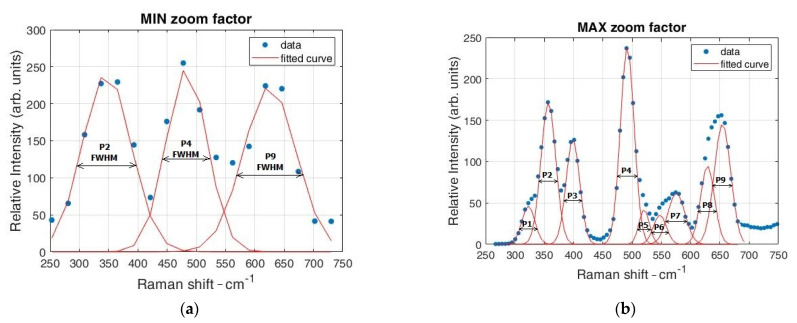
Calculation of the spectral resolution of the proposed system with a ZrO_2_ Raman spectrum using the Gaussian fit for each Stokes peak at different ZF. Note the difference in the number of peaks and fitted curves at the same spectral interval for minimum ZF (**a**) and maximum ZF (**b**).

We also tested the obtained resolution by measuring the linewidth for the band at 483.5 cm^−1^. We measured its linewidth at the minimal and maximal ZF by fitting the Gaussian profile (Figure 5a,b), resulting in 81.88 cm^−1^ at the minimum ZF and 27.62 cm^−1^ at the maximum ZF. By increasing the ZF, new Raman bands also appear, due to the increased resolution. 

Table 1 shows the Gaussian fitting parameters for each peak at the minimum and maximum ZF, together with their FWHM. It is evident from the linewidth of the characteristic peak at 483.5 cm^−1^ that the spectral resolution increased significantly, i.e., the spectral resolution calculated by the FWHM became more than three times larger.

Using the values $\Delta {\;\tilde{\nu }}_{slit}$ = 7.0 cm^−1^, $\Delta {\;\tilde{\nu }}_{line}$ = 19.0 cm^−1^ (measured by the reference spectrometer without bining factor), and fitted FWHM = 27.62 cm^−1^, by Equation (3), we found $\Delta {\;\tilde{\nu }}_{resolution}$ = 18.78 cm^−1^, which also agrees with previously estimated values. By using Equation (3) and FWHM = 81.88 cm^−1^ at the lowest ZF of the 483.5 cm^−1^ line, the estimated resolution, in this case, is $\Delta {\;\tilde{\nu }}_{resolution}$ = 78.42 cm^−1^, which is 4.17 times worse resolution in comparison with the ZF = 6. In general, most spectrometers are not routinely used at the limit of their resolution because of a number of different aberrations, so the slight differences from the expected value of six times more change in resolution when applaying ZF = 6 can occur. 

### 3.3. Photoluminescence Spectra

**Figure 6:**
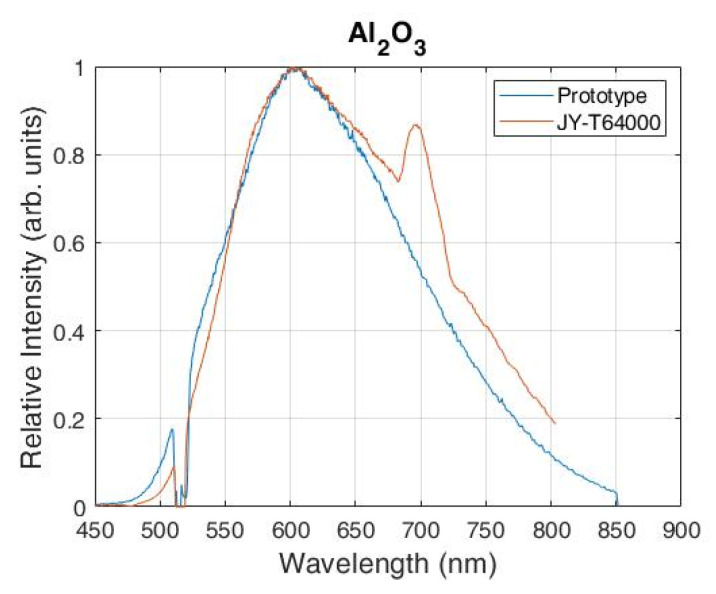
PL spectra of Al_2_O_3_ recorded with the proposed Raman spectrometer and the reference commercial JY-T64000 spectrometer.

The PL spectra of an Al_2_O_3_ shown in Figure 6 were recorded using the proposed system and the reference Raman spectrometer with an excitation laser of 532 nm. The exposure time was set to 3 s for both spectrometers. The ZF was set to 3× magnification so that the spectral interval from 450 to 850 nm could be recorded in a single shot. However, the total acquisition time for the same spectral interval using the reference spectrometer was around 30 times longer because the signal of each spectral interval of 20 nm width (on average) was collected separately (scaning multichanel mode) and then combined into one single spectrum. 

Such a procedure could result in artifacts, such as the one observed in the PL spectrum at wavelength of 690 nm shown in Figure 6, which does not exist in the PL spectrum of the Al_2_O_3_. This artifact could be explained by the spectral stitching in the reference spectrum, where the recording consists of 20–30 small sections of spectral intervals. These small sections of spectra were then merged, resulting in a spectrum with a large spectral interval. However, these small sections tend to carry variable background of the slope which changes during the recording. So, the combining procedure then had the wrong information on the PL intensity data at the beginning and the end of each section, leading to artificial peaks which occur after combining into a single spectrum.

## 4. Conclusions

A novel approach for Raman and PL spectroscopy based on a variable spectral resolution is described. The variable spectral resolution was achieved in Raman and PL spectra by using zoom optics in the collimating part of the monochromator. It was shown that the collimating optics’ different zoom factors directly affect the spectral resolution of the observed spectral interval. With the minimum ZF, the lowest spectral resolution with the largest spectral interval was achieved on the detector. On the contrary, with the maximum ZF, the largest spectral resolution with the smallest spectral interval was demonstrated on the detector. This approach allows the acquisition of Raman and PL spectra with significantly reduced time. Furthermore, the ability to vary the spectral range and resolution offers great flexibility in terms of applications and conditions of usage of such device, ranging from quick Raman measurements for the detection or confirmation of substances and broad-spectrum PL measurements to high-resolution measurements for detailed chemical and structural analysis.

To evaluate the performance of the proposed Raman spectrometer, a comparison was made with a high-grade laboratory Raman spectrometer (a reference spectrometer). The proposed approach outperforms the reference spectrometer in the PL measurement, in acquisition time as well as in avoiding the spectral artifacts produced by combining more spectral intervals into the single spectrum. Contrary to the spectral merging of the reference spectrometer, the variable spectral resolution spectrometer can be easily adjusted to collect the whole PL spectral interval at once. 

Finally, we showed that the Raman spectrometer with the zoom lens changes the spectral resolution and significantly reduces the acquisition time for Raman and PL spectra. However, there are already a couple of commercial spectrometers holding dual-mode acquisition for both Raman and PL. Simply by rotating the grating to reach blazing angles, a broad spectrum is obtained in a short time. Advantages of our approach include the two gratings and two fixed sizes of the spectrum on the detector, as well as the variable size of the spectrum at the detector with a single grating. This approach enables the recording of Raman and PL spectra within a single spectrometer. Moreover, our results show that such a spectrometer could be an efficient tool to inspect Raman and PL bands of unknown materials, and, thereafter, for the recording of wanted spectral interval at appropriate spectral resolution. 

The possibilities for further development of this approach are numerous. For example, this can be achieved by increasing the spectral resolution, using a zoom lens with higher ZF, using longer focus at highest ZF, and including a higher pixel number to the CCD/CMOS detector. On the software side, there is room for improvement in the ability to vary the resolution, data acquisition, and similar elements. We believe that the proposed prototype and this novel approach in spectroscopy will serve as a foundation for future research and open up a gateway for future inovative developments in the field.

## Figures and Tables

**Table 1:** Gaussian fit parameters for the obtained spectral peaks.

Curves	MIN ZF					MAX ZF			
	A	b [cm^−1^]	σ	FWHM		A	b [cm^−1^]	σ	FWHM
P1	n/a	n/a				46.1	306.34	11.14	26.22
P2	241.9	346.8	41.43	97.57		171.77	340.44	12.63	29.74
P3	n/a	n/a				127.1	382.25	11.59	27.29
P4	248.5	483.5	34.77	81.88		239.1	475.14	11.73	27.62
P5	n/a	n/a				42.2	503.97	8.76	20.63
P6	n/a	n/a				35.6	530.81	11.02	25.97
P7	n/a	n/a				61.97	558.99	14.61	34.41
P8	n/a	n/a				94.9	612.17	11.82	27.84
P9	223.8	625.45	45.13	106.28		145.5	637.68	13.97	32.9

## Data Availability

The data presented in this paper are available on reasonable request from the corresponding author.
